# An Ovarian Teratoma Mimicking Complicated Acute Appendicitis: A Case Report and Literature Review

**DOI:** 10.7759/cureus.36713

**Published:** 2023-03-26

**Authors:** Mihindukulasuriya Yvonne Presadini Pinto, Graham Hool

**Affiliations:** 1 Department of General Surgery, Royal Perth Hospital, Perth, AUS

**Keywords:** review of literature, surgical case reports, ovarian teratoma, mature cystic teratoma, right iliac fossa pain, complicated acute appendicitis

## Abstract

Acute appendicitis is one of the most common causes of right iliac fossa (RIF) pain in the younger population. However, multiple other pathologies presenting with RIF pain can mimic acute appendicitis. In the female gender, the differentials for RIF pain are broader. Multiple pathologies can present with similar symptomatology that can mimic acute appendicitis, leading to an incorrect diagnosis, unnecessary surgical interventions, and complications. In females of reproductive age, gynaecological causes can present similarly. Here, we present a case of an ovarian teratoma mimicking acute complicated appendicitis.

A female of reproductive age presented to our hospital with RIF pain of six days, associated with fever, nausea, vomiting, and anorexia. A clinical diagnosis of acute complicated appendicitis was suspected, and further imaging was arranged to confirm the diagnosis. Imaging showed a normal appendix with a right adnexal mass separated from the ovary, representing a teratoma. She underwent elective surgery for the excision of teratoma after further investigations. Ovarian teratomas are not a common mimicker of appendicitis. One should consider possible gynaecological causes as a differential for RIF pain. Due to the wide variety of differentials, when in doubt, especially in the female gender, further imaging should be considered for confirmation of diagnosis.

## Introduction

Acute appendicitis is one of the most common causes of right iliac fossa (RIF) pain in the younger population. Typical presentation includes periumbilical colicky abdominal pain migrating to RIF, associated with constitutional symptoms such as vomiting, nausea and anorexia. Acute appendicitis is primarily a clinical diagnosis [1]. However, less than half of the patients present with typical symptoms. A wide variety of differential diagnoses is available for RIF pain, especially in reproductive-age females such as endometriosis, adenomyosis, ectopic pregnancy and ovarian torsion. These could mimic acute appendicitis or acute complicated appendicitis.

In this report, we discuss a case of torsion of a large ovarian teratoma presenting as acute complicated appendicitis. Ovarian teratoma is not a common cause of RIF pain. Here, we will discuss the importance of thorough history taking, examination and appropriate investigations, including imaging, to make an accurate diagnosis and prevent unnecessary surgical Interventions. Our primary focus in this case report and literature review is on differentiating acute appendicitis from its mimickers.

This case report has been reported in line with the SCARE (Updating Consensus Surgical CAse REport) criteria [2].

## Case presentation

A 29-year-old nulliparous female presented to our emergency department with six days of non-migratory RIF pain associated with nausea, vomiting, fever and anorexia. She denied any vaginal bleeding or vaginal discharge. Her last menstrual period was one week ago. She was an otherwise healthy lady and was not on regular medications. On examination, she was febrile at 38.2° C and tender in the RIF with fullness. Her white cell count (WCC) (15.82 x 109/L) and neutrophil count (12.1 x 109/L) were high, with a markedly elevated C- reactive protein (CRP) (231 mg/L). Beta human chorionic gonadotropin (HCG) was negative. Due to ongoing fevers, a palpable mass in the RIF and markedly elevated CRP, the possibility of complicated appendicitis with an abscess or a phlegmon formation was suspected.

**Figure 1:**
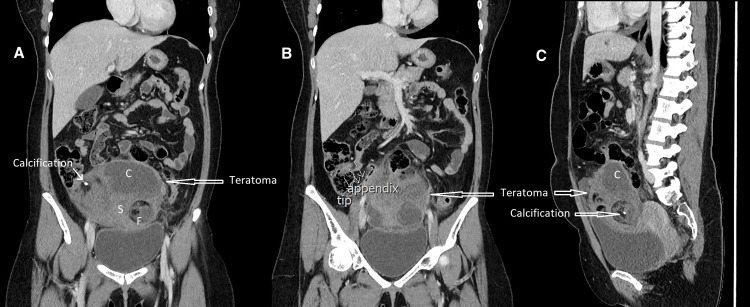
CT scan images of the abdomen and pelvis, with intravenous contrast, in the portal-venous phase A: Coronal view with large teratoma with calcification, solid (S) and cystic (C) areas and fatty tissue (F). B: Coronal view of the normal-looking appendix. Teratoma can be seen right next to the appendix. C: Sagittal view of the teratoma with cystic (C) and solid tissue, fatty tissue and calcification.

**Figure 2:**
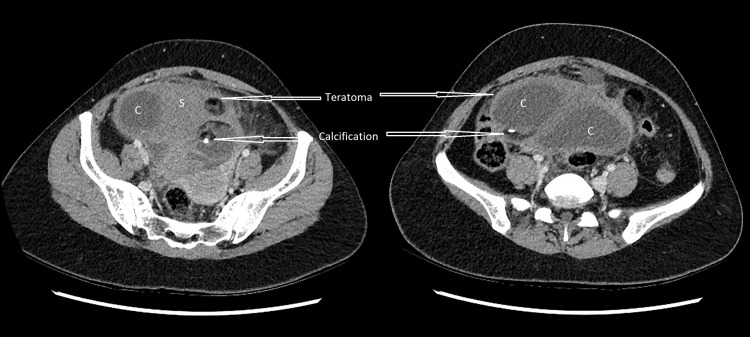
Axial images of CT abdomen Axial images of CT abdomen with intravenous contrast in the portal-venous phase. The two axial images show a teratoma with areas of fat, cystic (C) and solid (S) tissue and calcification. Surrounding fat stranding is visualised in both images.

She underwent a computed tomography (CT) scan to confirm the diagnosis. The CT scan showed a normal appendix and a 128 mm X 109 mm X 88 mm heterogeneous, mixed solid and cystic lesion within the pelvis (Figures 1, 2).

The lesion contained areas of fat and calcification, with some adjacent fat stranding, and was separately identified from the right ovary. The features of the cyst represented a teratoma. She was then transferred under the care of a gynaecologist for further management.

**Figure 3:**
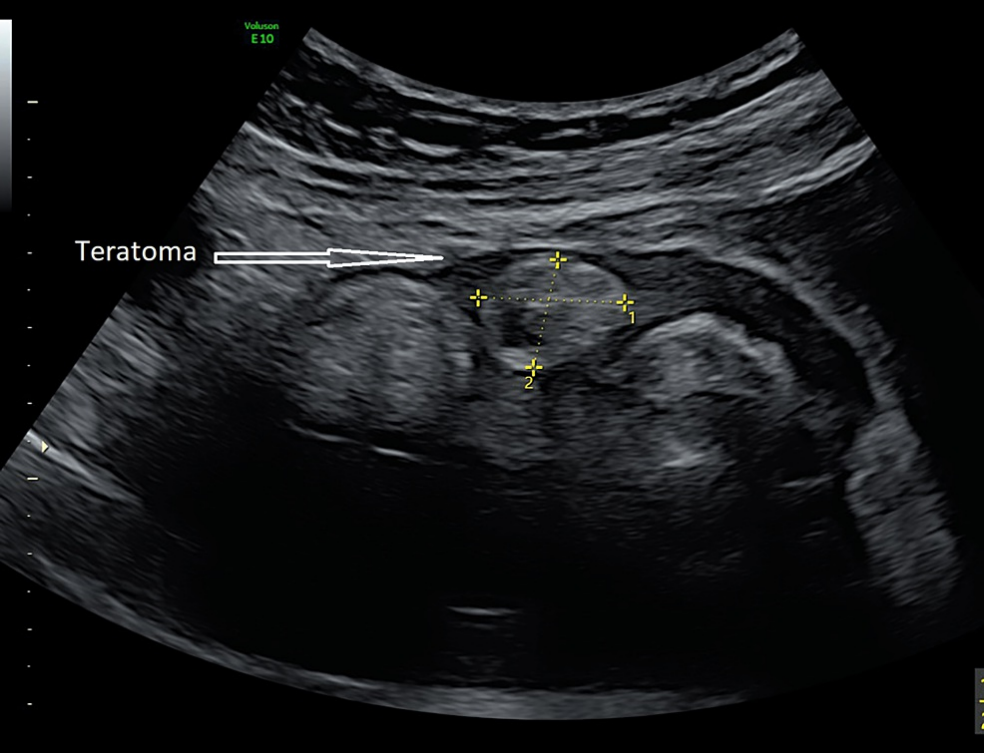
USS image of the pelvis showing a teratoma with fatty tissue USS: ultrasound scan

She underwent a transvaginal and a transabdominal ultrasound scan (USS) to characterise the cyst further (Figure 3).

The cyst volume was 537 ml with no internal vascularity. The right ovary was identified separately and adhered to the cyst. She had further blood tests, including tumour markers: alpha-fetoprotein, cancer antigen (CA) 19-9, carcinoembryonic antigen and CA 125. All the markers were within the normal range other than the mildly elevated CA 125.

**Figure 4:**
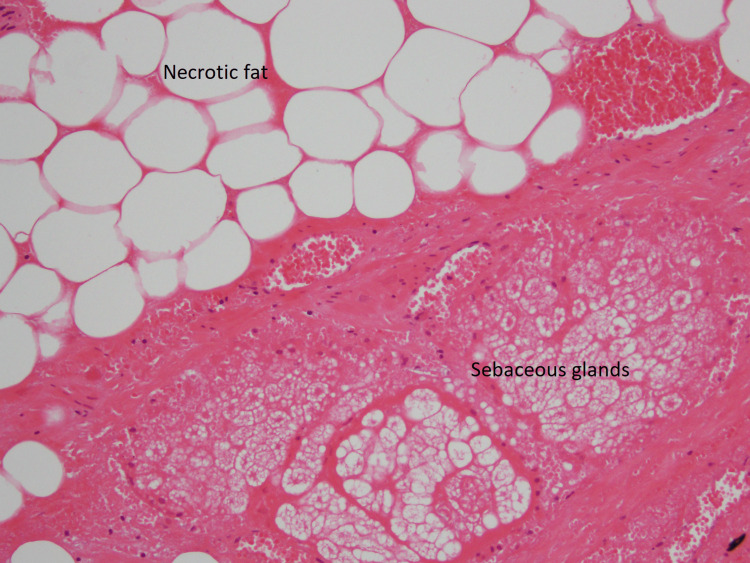
Histopathology of teratoma - medium magnification Histopathology- Medium magnification of tumour showing necrotic fat (above) and sebaceous glands (below).

**Figure 5:**
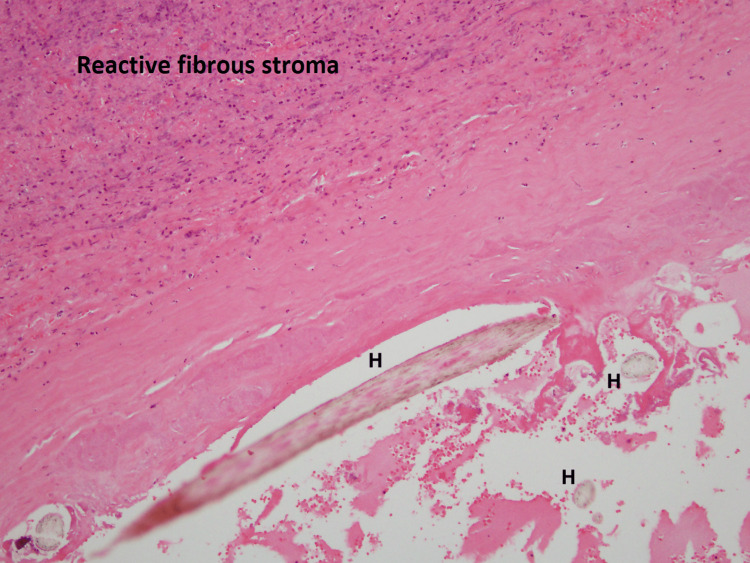
Histopathology of teratoma - medium magnification Histopathology - medium magnification showing reactive fibrous stroma (above) and fragments of hair shaft (H) material (below).

She underwent an uncomplicated elective laparoscopic surgery on day 6 from the presentation. The cyst (diameter of 15 cm) had adhered to the omentum, small bowel, pelvic sidewalls and anterior abdominal wall. The right ovary and tube were torted around the teratoma and necrosed. A right salpingo-oophorectomy was performed along with the excision of the cyst. The specimen was retrieved via an extended suprapubic incision. Histopathology showed haemorrhagic and largely infarcted fatty tissue with scattered hair shaft fragments, neuroglial tissue, cartilage and calcification (Figures 4, 5).

No immature or malignant elements were identified. She was sent home on the same day postoperative.

## Discussion

Acute appendicitis is one of the most common general surgical pathologies encountered in the emergency department. It is common in the age group 10 to 20 years [3] and is seen less often over the age of 50 years, accounting only for 15% of all acute abdomen cases presenting to the emergency department when compared to the younger population (30%) [4]. There is a slight predominance in males with a male-to-female ratio of 1.14: 1.00 [5]. As reported by Addiss et al. (1990), the crude incidence of acute appendicitis is 11 per 10,000 per year, with a lifetime risk of 8.6% for males and 6.7% for females [6]. According to the “Global burden of conditions requiring emergency surgery” study in 2010 by Stewart et al., acute appendicitis is the eighth most common cause of death in all surgical emergencies (including non-general surgical emergencies), with 0.5 deaths and 21 disability-adjusted life years per 100,000 population worldwide [4].

The typical presentation of acute appendicitis is colicky periumbilical pain, then migrating to RIF [7]. Pain is associated with constitutional symptoms such as nausea, vomiting, anorexia, and constipation. Pathophysiology of the initial periumbilical pain (visceral) is due to the embryological origin of the appendix from the midgut. Furthermore, the subsequent RIF pain is due to the irritation of the parietal peritoneum from the inflamed appendix. However, the typical presentation is seen in less than half of the cases and depends on the patient’s age, the position of the appendix [1] and other factors such as the immune status of the patients.

Periumbilical pain can be caused by many midgut origin viscera, leaving a wide variety of differentials in a patient presenting at the initial stage of the disease, for example, Meckel’s diverticulitis. Subsequent RIF pain can be caused by diseases in the terminal ileum (e.g., ileitis) and cecum (e.g., diverticulitis). Inflammation of the mesenteric lymph nodes resulting in mesenteric adenitis and fat necrosis of the epiploic appendages causing appendagitis epiploica are a few other causes of RIF pain. They can present with RIF pain with or without preceding periumbilical pain and constitutional symptoms, mimicking acute appendicitis. Gynaecological causes, especially in reproductive-age females, broaden the long differentials list for RIF pain. This is likely the reason for high negative appendicectomy rates in females aged 15 to 24, which is 2.5 times higher than the males of the same age [5].

There are scoring systems to guide the diagnosis of appendicitis, and the Alvarado score is one of the commonly used scores. One systematic review revealed that the Alvarado score overpredicts the probability in women; however, is a valuable tool to rule out the diagnosis of acute appendicitis at or below a score of five [8].

Teratoma is not a common mimicker of acute appendicitis. In our case, the patient presented with non-migratory RIF pain associated with nausea, vomiting and anorexia. She was not mid-cycle and denied any vaginal bleeding, vaginal discharge, or previous gynaecological history. Her serum beta-human chorionic gonadotropin (HCG) was negative. Considering this history, we ruled out many of the gynaecological causes. Our initial diagnosis was acute complicated appendicitis due to fever, six days of pain and the markedly elevated CRP level; hence, a CT scan was arranged to confirm the diagnosis and further characterise the appendicular complication. In this scenario, fever is likely caused by the necrosed ovary. The RIF pain is possibly explained by the increasing size of the teratoma exerting pressure symptoms, capsular distention of the teratoma, haemorrhage and infarction, and the torsion of the ovarian teratoma complex. Ovarian torsion is a gynaecological emergency that needs urgent attendance to restore the blood supply to salvage the ovary. In her case, the presentation was late with an already compromised blood supply, resulting in ovarian ischaemia and necrosis.

Teratomas can occur in other parts of the body; however, they are common in gonads. Ovarian teratomas are the most common germ cell tumour [9] and they contain tissue of ectodermal, mesodermal and endodermal origin. Ovarian teratomas are classified into mature cystic teratomas, immature teratomas and monodermal teratomas [9]. Our patient’s histopathology revealed a mature cystic teratoma, which has an incidence of 32.6% of all primary ovarian neoplasms [10]. These are benign neoplasms of the ovary with a probability of 0.8% undergoing malignant transformation [11].

Females of reproductive age can experience physiological pain secondary to ovulation, which could mimic appendicitis. Endometriosis, adenomyosis, pelvic inflammatory disease and retrograde spillage of menstrual blood are a few more causes that could mimic acute appendicitis. Other significant gynaecological causes are ectopic pregnancy and ovarian torsion, presenting as acute abdomen and will need emergency interventions. A thorough gynaecological history and examination, including a speculum examination in a reproductive-age female, is of value in differentiating such a pathology from acute appendicitis.

Jerusalem guidelines (2020) from the World Society of Emergency Surgery, recommend a customized approach to the diagnosis of acute appendicitis by constructing a diagnostic pathway based on age, gender and clinical symptomatology [12]. UpToDate guidelines recommend a pelvic examination and serum beta-HCG level (reproductive age) on females as a routine workup. In general, WCC with differentials and CRP levels are recommended; however, no cut-off level can safely confirm or eliminate the diagnosis of acute appendicitis [13]. CT is the preferred first-line imaging due to its high diagnostic accuracy. Moreover, USS is offered as the first line in pregnancy, paediatric population, and young females to avoid the radiation risk [13].

## Conclusions

Acute appendicitis is a clinical diagnosis. Multiple other pathologies can present similarly, mimicking acute appendicitis or acute complicated appendicitis and can cause diagnostic challenges as in this scenario. When assessing a patient, one must consider the many possible causes of RIF pain. For a female of reproductive age, gynaecological causes need to be considered as a differential diagnosis. Further investigations, including imaging, should be arranged in doubt. A thorough history, examination and appropriate investigations will help to prevent unnecessary surgical interventions.
